# The Potential Role of a Soluble γ-Chain Cytokine Receptor as a Regulator of IL-7-Induced Lymphoproliferative Disorders

**DOI:** 10.3390/ijms19113375

**Published:** 2018-10-28

**Authors:** Geona Kim, Yuna Jo, Byunghyuk Lee, Laraib Amir Ali, Boae Lee, Changwan Hong

**Affiliations:** 1 Department of Anatomy, Pusan National University School of Medicine, Yangsan 50612, Korea; rlarjsdk321@naver.com (G.K.); yoona30@naver.com (Y.J.); minimum91@naver.com (B.L.); laraib.meghjani@gmail.com (L.A.A.); balee2240@naver.com (B.L.); 2 Department of Rehabilitation Medicine, Pusan National University School of Medicine and Research Institute for Convergence of Biomedical Science and Technology, Pusan National University Yangsan Hospital, Yangsan 50612, Korea

**Keywords:** IL-7, sγc, lymphoma, cytokine

## Abstract

IL-7 is an essential, nonredundant growth factor for T and B cell generation and maintenance. While IL-7 deficiency results in lymphopenia, overexpression of IL-7 can cause neoplasia in experimental models. IL-7’s involvement in neoplasia has been appreciated through studies of IL-7 transgenic (Tg) mice models and human lymphoma patients. Since we recently found that a soluble form of the common γ-chain (γc) cytokine receptor (sγc) antagonistically regulates IL-7 signaling, IL-7 and sγc double-Tg mice were generated to investigate the effects of sγc overexpression in IL-7-mediated lymphoproliferative disorders (LPDs). The overexpression of sγc prevents IL-7Tg-induced abnormal increase of LN cell numbers and the development of splenomegaly, resulting in striking amelioration of mortality and disease development. These results suggest that modification of γc cytokine responsiveness by sγc molecules might control various γc cytokine-associated hematologic malignancy, and also provide an alternative view to approach antitumor therapy.

## 1. Introduction

IL-7 is one of the γc cytokines that are a major factor in T-cell development and differentiation [1]. On the other hand, the IL-7 has been identified as a supportive factor for several human lymphocyte malignancies, including Hodgkin’s and both B- and T-acute and -chronic lymphocytic leukemia [2,3]. IL-7 exerts its effect through interaction with the IL-7 receptor (IL-7R), which is composed of a unique α chain (IL-7Rα) and γc, whereby expression of IL-7Rα mainly determines the timing and extent of IL-7 signaling [1,4], since γc expression is presumed to remain unchanged on lymphocytes. This was proven by IL-7Tg animal models that were heterozygous with the IL-7Rα subunit and improved survival compared to wild-type (WT) IL-7Tg mice [5,6]. In addition, the heterozygote of signal transducers and activators of transcription (STAT) 5, the main component of JAK/STAT pathway of IL-7 signaling, showed a dramatic reduction in IL-7-induced mortality and tumor development [5]. γc is one of essential components for IL-7 signaling and is critical in lymphocyte development and homeostasis, while little information exists about the mechanisms of γc expression and regulation. 

IL-7 binding to IL-7R triggers two main pathways, STAT5 of the JAK/STAT pathway [7] and the phosphatidylinositol-3-kinase (PI3 kinase) pathway [8,9]. Both STAT5 and PI3 kinase have been implicated in cell-growth control and survival [10]. Consistent with the signaling pathways, constitutively overexpressed STAT5 transgenic animal models demonstrated that excessive cytokine signaling through their overexpression can develop lymphomas [11]. The role of STAT5 in lymphomagenesis was more supported by STAT5 heterozygote animal models that haploinsufficiency of the STAT5 transcription factor could significantly modify the consequence of IL-7 overexpression [5]. Interestingly, dysregulation of IL-7 signaling through retrovirally encoded γc has been considered a possible mechanism of leukaemogenesis after γc-gene therapy, because IL-7 levels are elevated in patients with severe combined immunodeficiency (SCID) [12]. However, the effects of γc transgenes in lymphomagenesis have not been directly evaluated yet.

Recently, we discovered sγc that is generated by alternative splicing of the γc pre-mRNA and that is highly released by activated T cells, negatively regulating γc cytokine signaling [13,14], resulting in the modulation of T-cell homeostasis and differentiation. It is interesting to speculate on whether sγc overexpression would ameliorate the development of IL-7-mediated lymphoproliferative disorders (LPDs) through the dampening of IL-7 signaling [15]. In this regard, our study revealed that sγc could significantly control IL-7-mediated LPDs. Furthermore, we suggest that the therapeutic potential of sγc could be applied and expanded to immune-associated diseases like autoimmune disease and infection.

## 2. Results and Discussion

### 2.1. IL-7-Mediated LPDs Are Regulated by sγc Expression Level

**Figure 1:**
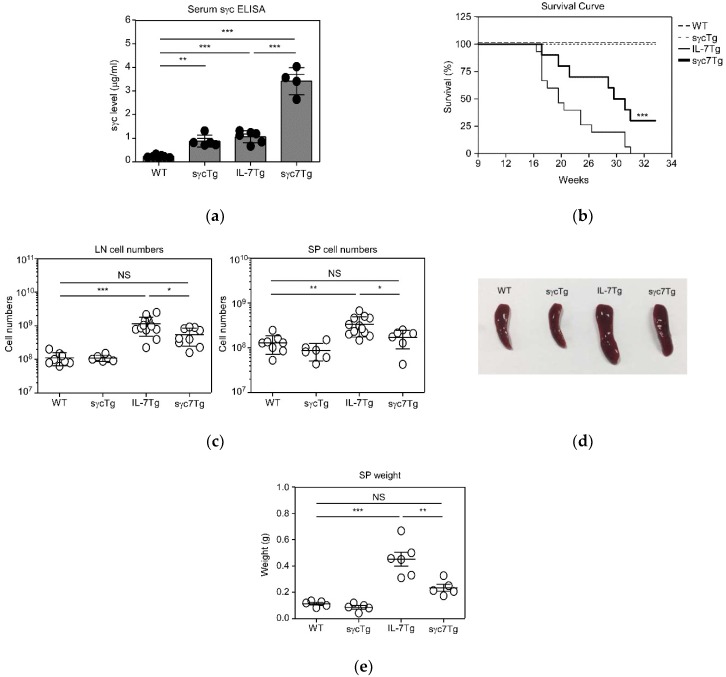
sγc levels affect IL-7-induced mortality. sγcTg mice were crossed with IL-7Tg mice and monitored weekly for lesions, node enlargement, and survival over eight months. (**a**) sγc levels in wild-type (WT), sγcTg, IL-7Tg, and sγc7Tg serum were measured by ELISA. (**b**) Survival of WT (*n* = 16), sγcTg (*n* = 14), IL-7Tg (*n* = 15), and sγc7Tg (*n* = 10) mice were monitored every week. (**c**) Total lymph-node (LN) and spleen (SP) cell numbers in indicated mice. Each symbol represents an indicated individual mouse. Horizontal lines indicate mean and SD. (**d**) Gross anatomy of spleens from the indicated mice. Picture shows representative spleen from *n* > 5 mice per group. (**e**) Summary of spleen weight from indicated mice. * *p* < 0.05, ** *p* < 0.01, *** *p* < 0.001, and NS mean not significant.

In previous reports, we demonstrated that sγc regulates γc cytokine signaling with the generation of sγc-overexpressing transgenic (sγcTg) mice [13,14,16]. We found with in vitro and in vivo analysis of γc cytokine responsiveness that an elevated level of sγc dramatically dampens IL-2, IL-7, and IL-15 signaling. Since IL-7Tg mice have been previously used to study IL-7 responsive tumor types and IL-7-mediated transformation [6], we generated sγc and IL-7 double-Tg (sγc7Tg) mice to investigate the effects of sγc overexpression in IL-7-mediated LPDs. First, we confirmed sγc levels in these mice, and found that sγc levels were significantly increased in sγcTg (878 ± 220 ng/mL) and sγc7Tg (3414 ± 499 ng/mL) compared to WT (227 ± 50 ng/mL) and IL-7Tg (1065 ± 230 ng/mL), respectively (Figure 1a). Comparing WT and IL-7Tg mice, the sγc level in IL-7Tg mice was dramatically increased (Figure 1a), indicating that an absolute amount of sγc may be proportional to the number of lymphocytes. Next, we examined whether the high level of sγc controlled IL-7Tg-induced death. IL-7 overexpression under the control of the immunoglobulin enhancer and promoter accelerated mortality compared to WT mice, with 100% mortality within six months of age, as described previously [6]. sγcTg mice displayed improved survival from IL-7-mediated death (Figure 1b), with survival well beyond eight months in 30% of sγc7Tg mice (*p* < 0.001). Moreover, while chronic overexposure of IL-7 resulted in an abnormally increased number of LN cells and splenomegaly development, sγc overexpression prevented IL-7Tg-induced abnormal increase of LN and spleen cell numbers (Figure 1c). Remarkably, sγc7Tg mice did not develop splenomegaly (Figure 1d,e). These findings indicate that sγc positively controls IL-7-induced LPDs. Altogether with the current results and our previous studies [13], we strongly expect that sγc overexpression protects against IL-7-mediated lymphomagenesis, and consequently reduces mortality by dampening excessive IL-7 signaling.

### 2.2. sγc Suppresses IL-7-Related Expansion of T Cells

**Figure 2:**
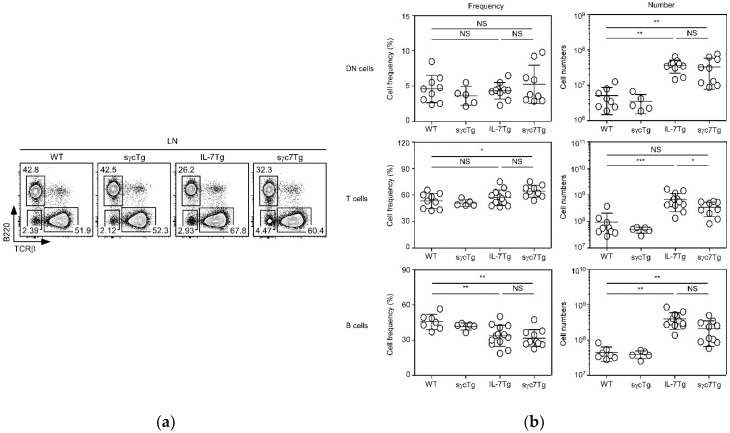
Frequency and total numbers of B, T, and B-T- (DN) cells in LN from the indicated mice. (**a**) Contour plots show TCRβ/B220 profiles and percentages of B, T and DN cells, respectively. (**b**) Summary of B, T, and DN cell frequency and numbers. Each symbol represents an indicated individual mouse. Horizontal lines indicate mean and SD. * *p* < 0.05, ** *p* < 0.01, *** *p* < 0.001, and NS mean not significant.

Since a decrease in the number of lymphocytes in sγc7Tg mice was observed, we next wanted to examine which immune cells are dominantly affected by sγc overexpression. While both B and T cells were vigorously expanded in IL-7Tg mice, sγc overexpression significantly and specifically reduced the expansion of T cells without alteration of their frequency and B:T ratio (Figure 2a,b). Indeed, while B cells become IL-7-independent with the termination of IL-7Rα expression after successful IgH rearrangement, T cells only start to be dependent on IL-7 upon TCR rearrangement. The inhibitory effect of sγc overexpression in T-cell expansion seems to be due to T-cell-specific IL-7 dependency. However, a numerical increase of both B (statistically significant) and T (statistically nonsignificant) cells was still observed in sγc7Tg mice compared to WT and sγcTg mice (Figure 2b). This may be due to the partial inhibitory effects on IL-7 signaling, or the mechanism in which these cells were transformed and acquired malignancy with chronic and strong IL-7 signaling. Consistent with previous studies [5,6], prolonged in vivo exposure of IL-7 leads to a preneoplastic lymphoproliferative state followed by the development of lymphoma. Thus, malignant cells among proliferative lymphocytes from sγc7Tg mice would proliferate regardless of the level of sγc. Indeed, this lymphomagenesis was proved in transplant experiments [6]. The IL-7-independent abnormal proliferation suggests that the inhibitory effect of sγc could be limited in IL-7-mediated lymphomagenesis.

**Figure 3:**
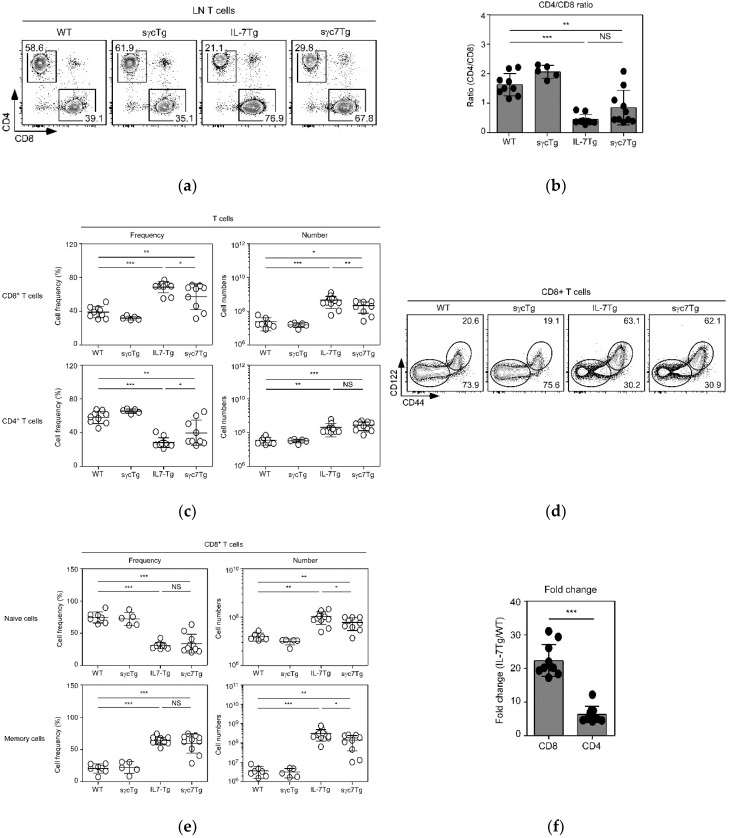
Analysis of CD4+ and CD8+ LN T cells. (**a**) Contour plots show CD4/CD8 profiles and percentages of CD4+ and CD8+ T cells, respectively. (**b**) CD4/CD8 ratio in the indicated mice. Each symbol represents an indicated individual mouse. Error bars represent mean and SD. (**c**) Summary of frequency and total numbers of CD4+ and CD8+ T cells in the indicated mice. Each symbol represents an indicated individual mouse. Horizontal lines indicate mean and SD. (**d**) Contour plots show CD44/CD122 profiles and percentages of CD44−CD122low naïve and CD44+CD122hi memory T cells, respectively. (**e**) Naïve- and memory-phenotype analysis of CD8+ T cells. Frequency and total numbers of naive and memory CD8+ T cells in the indicated mice were analyzed. Each symbol represents an indicated individual mouse. Horizontal lines indicate mean and SD. (**f**) Fold change of CD4+ and CD8+ T cells between IL-7Tg and WT mice. Each symbol represents an indicated individual mouse. Error bars represent mean and SD. * *p* < 0.05, ** *p* < 0.01, *** *p* < 0.001, and NS mean not significant.

The responsiveness of IL-7 in CD8+ and CD4+ T cells is quite distinct. In vivo administration of IL-7 to mice dramatically increases T cell numbers, especially CD8+ T cells [17]. Since CD8+ T-cell numbers are also dominantly increased in IL-7Tg mice [6,18], we next analyzed CD4 vs. CD8 profiles to confirm whether the sγc cell specifically regulates IL-7 signaling (Figure 3a). While CD4:CD8 ratios are significantly reduced in IL-7Tg mice compared to WT or sγcTg mice, these reductions are slightly restored by sγc overexpression (Figure 3b). The CD8+ T-cell-specific effect of sγc was definitely confirmed through analysis of change in cell numbers. The increased numbers of CD8+ T cells in IL-7Tg mice are significantly reduced in sγc7Tg mice; these reductions, however, were not observed in CD4+ T cells (Figure 3c). These data imply that sγc more specifically downregulates IL-7 signaling in CD8+ T cells than in CD4+ T cells, and the reduction in total T-cell numbers of sγc7Tg mice is due to a reduction in CD8+ T cell numbers. IL-7 plays a particularly important role in CD8+ T cell homeostasis, especially in the homeostasis of memory CD8+ T cells as well as naïve CD8+ T cells [19], while homeostatic proliferation of memory CD4+ T cells is independent of IL-7 [20]. Consistent with this, both naïve (CD44−CD122low) and memory (CD44+CD122hi) CD8+ T cell numbers are reduced in sγc7Tg mice compared to IL-7Tg mice (Figure 3d,e). Altogether with current studies and previous studies, we propose that sufficient IL-7 initially induces the proliferation of naïve CD8+ and CD4+ T cells, followed by differentiation to memory phenotype cells. Furthermore, since it has been shown that the survival and proliferation of memory CD8+ T cells, not CD4+ T cells, are dependent on IL-7 [18], CD8+ T cells are more dominantly expanded than CD4+ T cells in IL-7Tg mice (Figure 3f). Consequently, sγc relatively and more specifically suppresses the proliferation of CD8+ T cells. 

### 2.3. sγc Inhibits IL-7-Mediated Expansion of γδT and NKT Cells, but Not NK Cells

**Figure 4:**
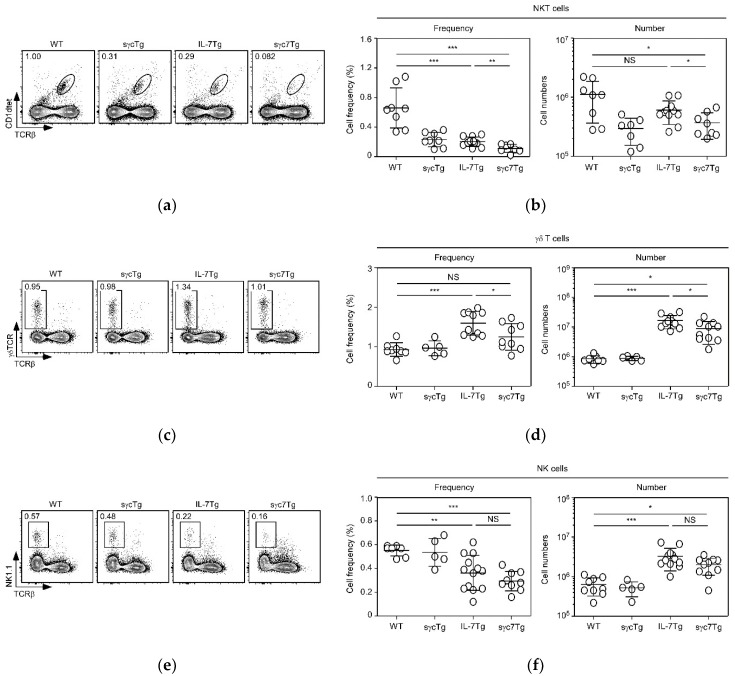
Frequency and total numbers of NKT, γδT, and NK cells in LN from indicated mice. Contour plots show (**a**) TCRβ/CD1dtet, (**c**) TCRβ/γδTCR, and (**e**) TCRβ/NK1.1 profiles and percentages of NKT, γδT, and NK cells, respectively. Summary of (**b**) NKT, (**d**) γδT, and (**f**) NK cell frequency and numbers. Each symbol represents an indicated individual mouse. Horizontal lines indicate mean and SD. * *p* < 0.05, ** *p* < 0.01, *** *p* < 0.001, and NS mean not significant.

We described the effects of sγc in IL-7-induced abnormal proliferation of conventional T cells. Since homeostatic expansion of nonconventional T cells, such as NKT and γδT cells, is dependent on IL-7 and IL-15 [21,22,23,24,25], we next analyzed the role of sγc in their proliferation. Consistent with our previous studies [14], we found that an elevated level of sγc reduces the frequency and numbers of NKT cells (Figure 4a,b). Interestingly, while significant reduction of NKT cell frequency was observed in IL-7Tg mice (Figure 4a), implying that is due to relative reduction by increased frequency of conventional T cells, NKT cell numbers in IL-7Tg were comparable to WT mice (Figure 4b). NKT cell frequency was more decreased in sγc7Tg mice compared to IL-7Tg mice (Figure 4b). This seems to be due to the synergetic effect of sγc overexpression as well as the relative reduction. This is further supported by the reduced number of NKT cells in sγc7Tg mice compared to IL-7Tg mice (Figure 4b). Thus, results indicate that the proliferation of NKT cells is IL-7-independent but sγc dependently inhibited. While it has been known that IL-15 plays a major role in NKT cell homeostasis [25,26], homeostasis of γδT cells is dependent on both IL-7 and IL-15 [22,23]. Consistently, the frequency and numbers of γδT cells were significantly increased in IL-7Tg mice; this increase, however, was markedly reduced by sγc overexpression (Figure 4c,d). Unlike NKT cells, these data demonstrate that γδT cells are responsible to IL-7 and could be developed into tumor cells, but γδT lymphomagenesis could be controlled by the level of sγc. In addition, we examined the frequency and numbers of NK cells (Figure 4e,f). Although homeostasis of NK cells is independent of IL-7 [27], the number of NK cells was increased in IL-7Tg mice (Figure 4f). This seems to be due to a bystander effect by proliferating other cells, rather than a direct effect of IL-7, since sγc7Tg mice did not show significant alteration in NK cell numbers (Figure 4f).

Our studies suggest that sγc contributes to the suppression of IL-7-induced lymphomagenesis. Consistent with our results, a study using a different target also suggests that regulation of IL-7 responsiveness in lymphocytes controls IL-7-induced lymphomagenesis [5]. IL-7 is one of the γc cytokines, including IL-2, IL-4, IL-9, IL-15, and IL-21, which share γc as a signaling subunit of their receptors. Many reports have also indicated that these γc cytokines are involved in neoplasia [15]. In terms of IL-2-associated neoplasia, certain T, B, monocytic, and even granulocytic leukemia cells express IL-2Rα. For example, human T-cell lymphotrophic virus I (HTLV-I)-associated adult T-cell leukemia cells constitutively express IL-2Rα [28]. IL-4 amounts are usually elevated in human cancer patients. IL-4 knockout mice are more resistant to tumor challenge than IL-4-competent mice [29]. Furthermore, the increase of IL-4 levels in tumor environments and the upregulation of the IL-4 receptor (IL-4R) on tumor cells have been long observed [30]. The incidence of T lymphoblastic lymphomas and large-B-cell lymphomas, induced by the retroviral transfer of the nucleophosmin-anaplastic lymphoma kinase (NPM-ALK) fusion gene product into bone-marrow cells, is increased in mice that transgenically express IL-9 [31]. Overexpression of IL-15 frequently develops T-NK lymphocytic leukemia [32]. Furthermore, IL-15 has been shown to promote leukemia-cell survival and/or proliferation [33]. A study using human myeloma cell lines showed the persistent expression of IL-21R on malignant plasma cells and potent antiapoptotic effects of IL-21, suggesting that IL-21 is associated with malignancy of multiple myeloma (MM) [34]. Furthermore, it has been reported that IL-21 works as a potent growth factor for certain hematological tumors, such as MM, Hodgkin’s lymphoma, anaplastic large-cell lymphoma, and cutaneous T-cell lymphoma, including Sézary syndrome [15].

Many reports and clinical studies have reported that γc cytokines are closely linked to the growth of tumor cells, especially hematologic malignancy [15]. It is easy to think that the approach of targeting a single γc cytokine cannot efficiently control hematologic malignancy, since other γc cytokines compensate for the loss of a targeted γc cytokine and serve as a tropic factor for lymphoma. Therefore, since “integrative control” of all γc cytokines is required for the efficient treatment of hematologic malignancy, we reason that sγc, as demonstrated in this study, would be a powerful biological drug for antitumor immunotherapy. Although there may be concerns about side effects caused by blocking all γc cytokine signaling, we would like to point out that sγc suppresses but not completely abolishes cytokine signaling, unlike a neutralizing antibody. This is in line with our observation that T-cell numbers are reduced but not completely rescued in sγc 7Tg mice to those of WT mice (Figure 2b), and IL-2 and IL-15 signaling is reduced but not absent in the presence of sγc [13,14,16]. Moreover, we propose that the modification of sγc to a multimer, which may improve affinity to γc cytokine receptors, would induce a more significant antitumor effect. 

## 3. Materials and Methods 

### 3.1. Animal

C57BL/6 (B6) mice were obtained from Orient Bio Inc. (Sungnam, South Korea). IL-7Tg mice, which were under the control of a murine H chain Ig enhancer (Eμ) and a human H chain Ig promoter (Pμ) [6], were generated by P. Leder (Harvard Medical School, Boston, MA, USA) and kindly provided by N. Abraham (University of British Columbia, Canada). sγcTg mice, which specifically overexpress sγc cDNA in T cells under the control of human CD2 (hCD2) enhancer–promoter [13], were bred with IL-7Tg mice to generate sγc- and IL-7-overexpressing mice. All mice were maintained in the specific pathogen-free animal facility of Pusan National University School of Medicine. All animal experiments and protocols were approved by the Pusan National University Institutional Animal Care and Use committee (PNU-2018-1850, 21 March 2018). 

All groups contained both female and male mice and were used at 8–10 weeks of age for FACS and anatomic analysis. Mice survival was accessed with spontaneous death.

### 3.2. Flow Cytometry Analysis

The cells were collected from LN and SP, and analyzed via FACS Canto or FACS Aria I. The data were analyzed using FlowJo version 10 (Version 10, Tree Star, Ashland, OR, USA). Antibodies with the following specificities were used for staining: CD8α (53–6.7), CD44 (IM7), NK1.1 (PK136), TCRβ (H57–597), γδTCR (GL3), and CD122 (IL-2R), all from BioLegend (San Jose, CA, USA); B220 (RA3–6B2) from BD Biosciences (San Jose, CA, USA); CD4 (GK1.5) from Thermo Fisher (Waltham, MA, USA), fluorochrome-conjugated CD1d tetramers loaded with PBS-567 and unloaded controls were obtained from the NIH tetramer facility (Emory University, Atlanta, GA, USA). Antimouse CD16/32 (2.4G2; BioLegend, San Jose, CA, USA) blocks nonspecific binding of antibodies. 

### 3.3. Detection of sγc Levels

Serum sγc was detected in a sandwich ELISA using a polyclonal anti-γc antibody (R&D system, Minneapolis, MN, USA) as the capture antibody, and a biotin-conjugated monoclonal anti-γc antibody (4G3; BD Biosciences) as the detection antibody. Recombinant sγc protein was used in standard curve.

### 3.4. Statistical Analysis

Statistical differences were analyzed using Student’s two-tailed *t*-test for comparisons between two groups, and one-way analysis of variance (ANOVA) for comparisons between more than two groups. Survival curve comparisons between two groups were performed with a nonparametric Mann–Whitney test. Calculations were performed by using Graph Pad Prism software Prism 5.0 (GraphPad Software, La Jolla, CA, USA) Statistical significance was determined as the following indications: * *p* < 0.05, ** *p* < 0.01, *** *p* < 0.001, and NS (not significant).

## Abbreviations

IL	Interleukin
TCR	T-cell receptor
NK	Natural killer
SCID	Severe combined immunodeficiency
